# Differences in Telemedicine, Emergency Department, and Hospital Utilization Among Nonelderly Adults with Limited English Proficiency Post-COVID-19 Pandemic: a Cross-Sectional Analysis

**DOI:** 10.1007/s11606-023-08353-7

**Published:** 2023-08-17

**Authors:** Eva Chang, Teaniese L. Davis, Nancy D. Berkman

**Affiliations:** 1 Advocate Aurora Research Institute, Advocate Health, 945 N. 12th St., Milwaukee, WI 53233 USA; 2 Center for Research and Evaluation, Kaiser Permanente Georgia, Atlanta, GA USA; 3 RTI International, Research Triangle Park, Retired, NC USA

**Keywords:** language, disparities, telemedicine, healthcare utilization, COVID-19.

## Abstract

**Background:**

The unprecedented use of telemedicine during the COVID-19 pandemic provided an opportunity to examine its uptake among individuals with limited English proficiency (LEP).

**Objective:**

To assess telemedicine use among nonelderly adults with LEP and the association between use of telehealth and emergency department (ED) and hospital visits.

**Design:**

Cross-sectional study using the National Health Interview Survey (July 2020–December 2021)

**Participants:**

Adults (18–64 years), with LEP (*N*=1488) or English proficiency (EP) (*N*=25,873)

**Main Measures:**

Telemedicine, ED visits, and hospital visits in the past 12 months. We used multivariate logistic regression to assess (1) the association of English proficiency on having telemedicine visits; and (2) the association of English proficiency and telemedicine visits on having ED and hospital visits.

**Key Results:**

Between July 2020 and December 2021, 22% of adults with LEP had a telemedicine visit compared to 35% of adults with EP. After controlling for predisposing, enabling, and need factors, adults with LEP had 20% lower odds of having a telemedicine visit than adults with EP (*p*=0.02). While English proficiency was not associated with ED or hospital visits during this time, adults with telemedicine visits had significantly greater odds of having any ED (aOR: 1.80, *p*<0.001) and hospital visits (aOR: 2.03, *p*<0.001) in the past 12 months.

**Conclusions:**

While telemedicine use increased overall during the COVID-19 pandemic, its use remained much less likely among adults with LEP. Interventions targeting structural barriers are needed to address disparities in access to telemedicine. More research is needed to understand the relationship between English proficiency, telemedicine visits, and downstream ED and hospital visits.

**Supplementary Information:**

The online version contains supplementary material available at 10.1007/s11606-023-08353-7.

## INTRODUCTION

In the USA, over 25 million people have limited English proficiency (LEP), or speak English “less than very well.”^1^ LEP is associated with low English literacy overall and low health literacy, increasing the risk of poorer access and use of healthcare, including being less likely to have doctor’s visits and more likely to have preventable emergency department (ED) visits.^2–9^ During the pandemic, more than 50% of adults with LEP reported delayed or forgone healthcare, compared with less than 20% prior to the pandemic, likely reflecting significant barriers to access worsened by the pandemic.^9,10^

The COVID-19 pandemic accelerated the digital transformation of healthcare by inducing clinicians, practices, and healthcare systems to shift much of their clinical care from in-person to virtual telemedicine visits, starting in March 2020. Several studies have shown the growth of telemedicine among patients and healthcare providers since the start of the pandemic.^11–14^ At its peak in April 2020, telemedicine constituted 42% of ambulatory visits.^13^

Many studies have found disparities in telemedicine use among underserved and vulnerable populations.^12,15–20^ Adults with LEP are particularly vulnerable to being left behind by the digital divide and telemedicine because structural barriers related to technology use (e.g., access to internet-connected devices) and language barriers often compound one another.^15,21^ Since the onset of the pandemic, studies of specific healthcare systems found that LEP patients were 16–37% less likely to have a telemedicine visit than EP patients.^17,18^

The impact of access to telemedicine on the use of other healthcare services, including ED visits and hospital visits, is unclear. Findings from the limited literature examining the relationship between telemedicine and other healthcare services have been mixed.^19,22–26^ To our knowledge, no national studies have examined the relationships between English proficiency, telemedicine use, and ED and hospital visits.

Given the dramatic increase in the use of telemedicine in healthcare, there is a pressing need to understand telemedicine use among adults with LEP. Using a nationally representative sample, our objective was to assess telemedicine use among nonelderly adults with LEP after the pandemic onset (July 2020 to December 2021). We aimed to examine (1) the use of telemedicine among adults with LEP compared to adults with English proficiency (EP) and (2) whether having telemedicine visits modified the relationship between English proficiency and ED and hospital visits. We hypothesized that adults with LEP were less likely than adults with EP to have telemedicine visits, and that having telemedicine visits would modify the relationship between English proficiency and ED and hospital visits. Understanding the uptake of technology among adults with LEP is needed to ensure equitable access to healthcare services.

## METHODS

### Data and Sample

This study combined data from the 2020 and 2021 National Health Interview Surveys (NHIS), a nationally representative, cross-sectional household survey of the civilian, noninstitutionalized US population.^27,28^ Between July 2020 and April 2021, interviews were first conducted over the telephone, with in-person follow-up to complete interviews.^29,30^ In May 2021, interviews returned to their standard process (in-person with a telephone follow-up).^30^ The final adult response rate was 48.9% for 2020 and 50.9% in 2021.^29,30^ After excluding respondents with missing values (1.1% of the sample), our analytic sample included nonelderly, White, Hispanic, and Asian adult respondents interviewed between July 2020 and December 2021 (*n*=27,361). Less than 0.4% of Black and Other race respondents identified as LEP; these groups were excluded to focus the analyses on the healthcare utilization of the LEP population. This study used publicly available, de-identified data and was deemed to not be human subject research by the Advocate Aurora Health Institutional Review Board.

### Measures

We assessed types of healthcare utilization during the past 12 months based on self-report measures: having any telemedicine visits, any ED visits, and any hospital visits. All outcomes were dichotomous. To measure telemedicine visits, respondents were asked whether they “had an appointment with a doctor, nurse, or other health professional by video or by phone.”^29^ Other utilization outcomes were constructed from a question asking respondents how many times they had visited the ED (any ED visits), and if they had been hospitalized overnight (any hospital visits). Full question texts can be found in Appendix A.

The primary independent variable was English proficiency. Respondents were categorized as LEP if their interview was conducted in Spanish, both English and Spanish, or some other language; interviews with EP respondents were conducted in English only. Following the Andersen model to account for factors that may affect healthcare utilization,^31^ we controlled for predisposing, enabling, and need factors. Predisposing factors included age (18–29, 30–29, 40–49, 50–64 years), sex (male, female), and race and ethnicity (non-Hispanic White, Hispanic, non-Hispanic Asian). Need factors included health status (excellent/very good/good, fair/poor), disability (yes, no), and any 1 of 6 common chronic conditions (arthritis, cancer, congestive heart disease, diabetes, hypertension, high cholesterol). Enabling factors included education (some college or higher, high school diploma, less than high school diploma), family income (<100% of federal poverty level [FPL], 100-199% FPL, ≥200% FPL), insurance coverage (private, public/other, uninsured), usual place of care (yes, no), and place of residence (metropolitan or nonmetropolitan).

### Statistical Analysis

We used the *χ*^2^ test to compare differences in predisposing, enabling, and need factors by English proficiency. We also used the *χ*^2^ test to examine differences in telemedicine visits between and across survey quarters. We then conducted two analyses using multivariate logistic regression models.

The first analysis examined the association between English proficiency and having a telemedicine visit, and whether the relationship changed with the systematic inclusion of predisposing, enabling, and need factors. Model 1 included only predisposing factors, model 2 added need factors, and model 3 further added enabling factors.

The second analysis assessed the association between English proficiency and having any ED visits and any hospital visits. We first estimated models controlling for predisposing, need, and enabling factors. We then re-estimated the models adding telemedicine visits as an independent variable. In a third specification, we added an interaction term to assess whether having telemedicine visits moderated the association between English proficiency and other types of healthcare utilization. Lastly, we conducted a sensitivity analysis to assess whether the interaction between English proficiency and utilization differed by Hispanic ethnicity and Asian race, compared to White race.

All analyses were conducted using Stata version 17.0 (Stata Corporation, College Station, TX) and used statistical methods to account for the complex survey design (i.e., weighting). Two-sided *p*<0.05 was considered statistically significant.

## RESULTS

This study included 27,361 nonelderly, adult respondents, representing over 163 million people nationally; 7.5% were identified as LEP. Table 1 shows descriptive characteristics of English proficiency. Adults with LEP were significantly more likely to be middle-aged and Hispanic, have worse health, not have a high school diploma, have a family income below <200% FPL, have public insurance or be uninsured, live in a metropolitan area, and not have a usual place of care. Similar percentages of adults with LEP and EP had a disability and at least one chronic condition.

**Table 1:** Characteristics of Nonelderly US Adults, by English Proficiency, National Health Interview Survey, July 2020–December 2021

	Total (*N*=27,361)		English-proficient (*N*=25,873)		Limited English-proficient^a^ (N=1488)		*P*-value
	%	(95% CI)	%	(95% CI)	%	(95% CI)	
Age (years)							p<0.001
18–29	25.4	(24.6, 26.2)	26.0	(25.2, 26.9)	17.1	(14.7, 19.8)	
30–39	21.9	(21.3, 22.5)	21.8	(21.2, 22.4)	23.1	(20.8, 25.6)	
40–49	20.8	(20.2, 21.4)	20.3	(19.7, 20.9)	27.2	(24.4, 30.1)	
50–64	31.9	(31.2, 32.7)	31.9	(31.1, 32.7)	32.6	(29.7, 35.7)	
Female	50.3	(49.6, 51.0)	49.9	(49.2, 50.7)	55.0	(51.9, 58.0)	p=0.002
Race/ethnicity							p<0.001
White, non-Hispanic	70.2	(68.4, 71.9)	75.7	(74.3, 77.1)	1.8	(1.3, 2.6)	
Hispanic	22.4	(20.8, 24.1)	16.9	(15.7, 18.2)	90.9	(88.5, 92.9)	
Asian, non-Hispanic	7.4	(6.7, 8.1)	7.4	(6.7, 8.1)	7.3	(5.5, 9.6)	
Health status							p<0.001
Excellent/very good/good	89.8	(89.3, 90.3)	90.5	(90.1, 91.0)	80.6	(78.0, 82.9)	
Poor/fair	10.2	(9.7, 10.7)	9.5	(9.0, 9.9)	19.4	(17.1, 22.0)	
Has a disability	5.7	(5.3, 6.0)	5.7	(5.3, 6.0)	5.5	(4.2, 7.2)	p=0.85
Has ≥1 chronic condition	40.8	(40.0, 41.6)	40.8	(40.0, 41.6)	40.9	(38.0, 43.9)	p=0.94
Education							p<0.001
Some college or more	63.9	(62.8, 65.0)	67.2	(66.2, 68.2)	23.1	(20.2, 26.2)	
High school graduate	27.1	(26.2, 28.0)	26.9	(26.0, 27.8)	29.4	(26.5, 32.5)	
<High school	9.0	(8.4, 9.7)	5.9	(5.5, 6.4)	47.5	(43.7, 51.4)	
Family income (% FPL)							p<0.001
≥200%	75.0	(74.0, 76.0)	78.3	(77.4, 79.1)	34.9	(32.0, 37.9)	
100–199%	15.7	(15.0, 16.4)	14.1	(13.4, 14.7)	35.6	(32.8, 38.5)	
<100%	9.3	(8.7, 9.9)	7.7	(7.2, 8.2)	29.5	(26.5, 32.7)	
Insurance							p<0.001
Private	70.9	(69.9, 72.0)	74.1	(73.2, 75.0)	31.4	(28.2, 34.7)	
Public	16.2	(15.5, 17.0)	15.6	(14.9, 16.3)	24.3	(21.4, 27.3)	
Uninsured	12.8	(12.0, 13.6)	10.3	(9.7, 10.9)	44.4	(40.7, 48.1)	
Location							p=0.009
Metropolitan	87.0	(85.6, 88.3)	86.5	(85.1, 87.7)	94.3	(88.6, 97.2)	
Nonmetropolitan	13.0	(11.7, 14.4)	13.5	(12.3, 14.9)	5.7	(2.8, 11.4)	
Has a usual place of care	86.3	(85.7, 86.9)	87.0	(86.4, 87.6)	77.1	(74.1, 79.7)	p<0.001

Abbreviations: *FPL* federal poverty level

Data source: National Center for Health Statistics, National Health Interview Survey, July 2020–December 2021

^a^Respondents with limited English proficiency were identified using the language they used to respond to the survey

^b^Chronic conditions included arthritis, cancer, congestive heart disease, diabetes, hypertension, or high cholesterol

### Rates of Telemedicine Visits and Other Healthcare Utilization

**Figure 1:**
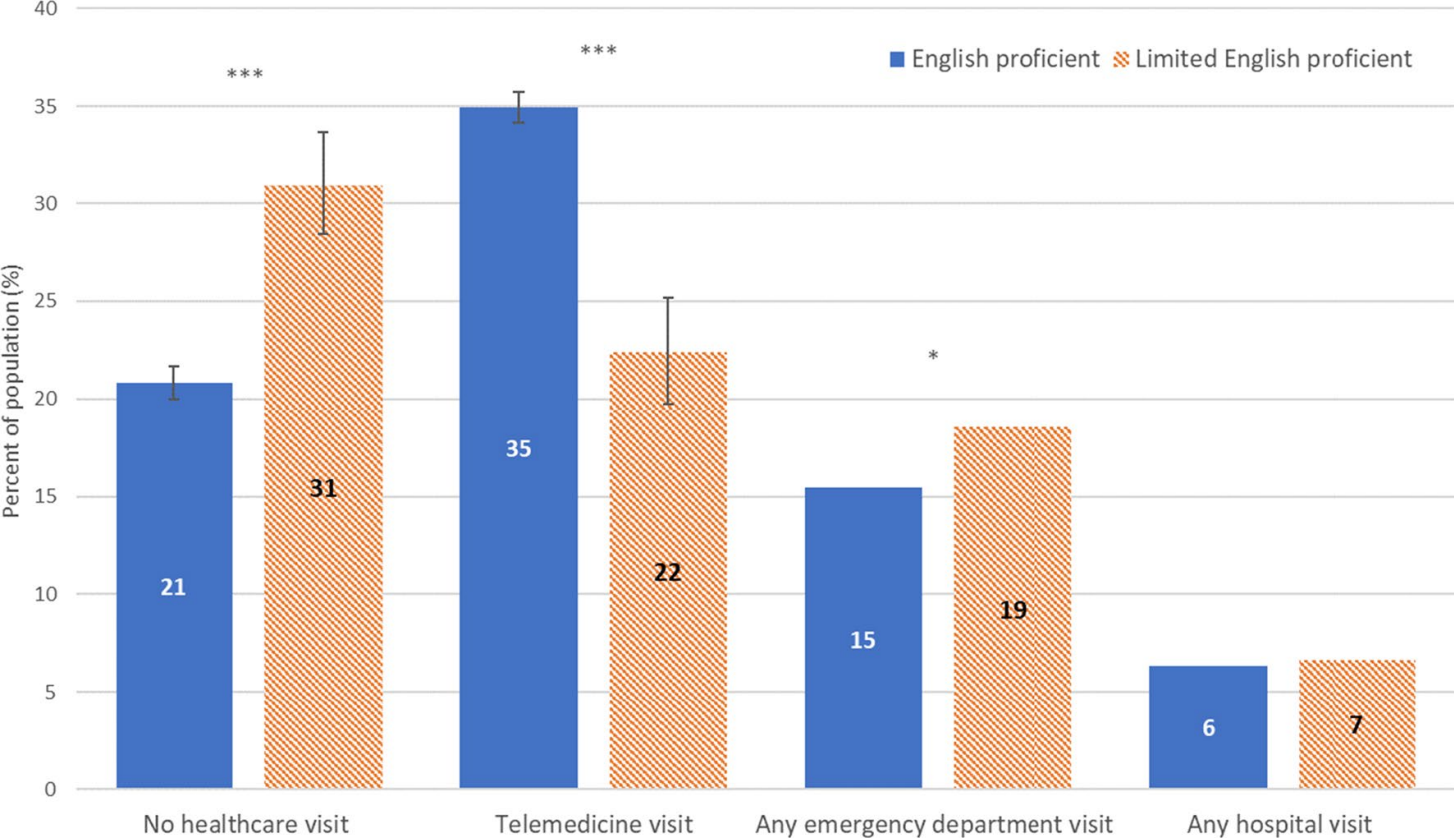
Healthcare utilization in the past 12 months among nonelderly US adults, by English proficiency, National Health Interview Survey, July 2020–December 2021. *p<0.05, **p<0.01, ***p<0.001. Error bars indicate 95% confidence intervals. Data source: National Center for Health Statistics, National Health Interview Survey, July 2020–December 2021.

Figure 1 presents the rates of healthcare utilization by English proficiency reported from July 2020 to December 2021. Compared to adults with EP, in the past 12 months, a significantly smaller percentage of adults with LEP reported having any telemedicine visits (22% vs. 35%, *p*<0.001). In contrast, compared to adults with EP, a larger percentage of adults with LEP reported having any ED visits (19% vs. 15%, *p*=0.01), while a similar percentage reported having any hospital visits (7% vs. 6%, *p*>0.05).

**Figure 2:**
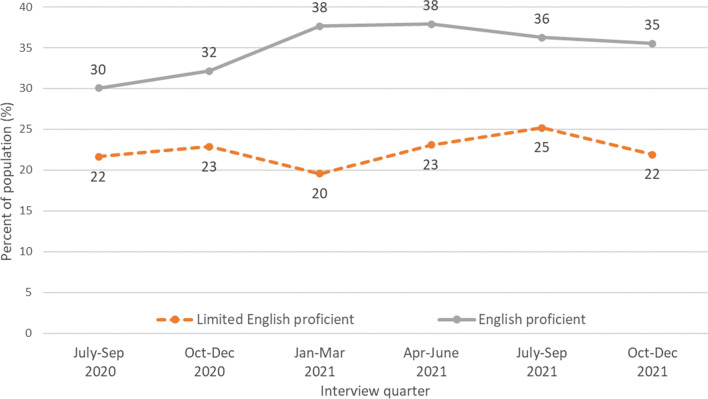
Any telemedicine visits in the past 12 months by interview quarter among nonelderly US adults, National Health Interview Survey, July 2020–December 2021. Data source: National Center for Health Statistics, National Health Interview Survey, July 2020–December 2021.

Figure 2 presents rates of telemedicine visits in the past year by English proficiency reported in each interview quarter. The percentage of adults with EP who had a telemedicine visit during the past year increased by 18% during the period (30% in quarter 3 of 2021 to 35% in quarter 4 of 2022). In contrast, the percentage of adults with LEP who had a telemedicine visit remained virtually constant, increasing from 21.6 to 21.9%, over the same period. In each survey quarter throughout the period, compared to adults with EP, a significantly smaller percentage of adults with LEP reported having telemedicine visits in the past 12 months (all *p*<0.05).

### Factors Associated with Telemedicine Visits

Table 2 shows the association of English proficiency on telemedicine visits, controlling for predisposing, need, and enabling factors. Controlling for predisposing factors only (model 1), adults with LEP had much lower odds of having a telemedicine visit (aOR: 0.56, *p*<0.001). After controlling for need as well as predisposing factors (model 2), the negative relationship between LEP and telemedicine visits remained large and virtually unchanged (aOR: 0.52, *p*<0.001). Finally, in model 3, fully adjusting for predisposing, need, and enabling factors, the association between LEP and telemedicine is attenuated but continues to be statistically significant (aOR: 0.80, *p*=0.02).

**Table 2:** Multivariable Associations with Any Telemedicine Visits Among Nonelderly US Adults, National Health Interview Survey, July 2020–December 2021

	Model 1		Model 2		Model 3	
	aOR (95% CI)	*p*-value	aOR (95% CI)	*p*-value	aOR (95% CI)	*p*-value
English proficiency						
English-proficient	Ref.		Ref.		Ref.	
Limited English-proficient	0.56 (0.47, 0.67)	<0.001	0.52 (0.44, 0.62)	<0.001	0.80 (0.66, 0.96)	0.02
Predisposing factors						
Age						
18–29	Ref.	Ref.	Ref.		Ref.	
30–39	1.28 (1.17, 1.41)	<0.001	1.15 (1.05, 1.26)	0.003	1.09 (0.99, 1.20)	0.07
40–49	1.43 (1.30, 1.57)	<0.001	1.10 (1.00, 1.21)	0.06	1.01 (0.91, 1.12)	0.86
50–64	1.54 (1.41, 1.69)	<0.001	0.94 (0.85, 1.04)	0.25	0.85 (0.77, 0.94)	0.002
Sex						
Male	Ref.	Ref.	Ref.		Ref.	
Female	1.76 (1.66, 1.87)	<0.001	1.81 (1.71, 1.93)	<0.001	1.72 (1.61, 1.83)	<0.001
Race/ethnicity						
Non-Hispanic White	Ref.	Ref.	Ref.		Ref.	
Hispanic	0.85 (0.78, 0.94)	0.001	0.86 (0.78, 0.94)	0.002	0.97 (0.87, 1.07)	0.48
Non-Hispanic Asian	0.78 (0.68, 0.89)	<0.001	0.86 (0.75, 0.98)	0.03	0.75 (0.65, 0.86)	<0.001
Need factors						
Health status						
Excellent/very good/good			Ref.		Ref.	
Poor/fair			1.71 (1.54, 1.91)	<0.001	1.98 (1.77, 2.22)	<0.001
Has a disability						
No			Ref.		Ref.	
Yes			1.59 (1.38, 1.83)	<0.001	1.70 (1.47, 1.97)	<0.001
Has ≥1 chronic condition						
No			Ref.		Ref.	
Yes			1.98 (1.84, 2.12)	<0.001	1.91 (1.78, 2.06)	<0.001
Enabling factors						
Education						
Some college or more					Ref.	
High school diploma					0.67 (0.62, 0.73)	<0.001
Less than high school diploma					0.53 (0.45, 0.64)	<0.001
Family income (% FPL)						
≥200%					Ref.	
100–199%					0.87 (0.79, 0.97)	0.010
<100%					0.83 (0.73, 0.95)	0.006
Insurance						
Private					Ref.	
Public/other					1.30 (1.17, 1.43)	<0.001
Uninsured					0.45 (0.39, 0.52)	<0.001
Location						
Metropolitan					Ref.	
Nonmetropolitan					0.58 (0.51, 0.66)	<0.001
Usual place of care						
Yes					Ref.	
No					0.44 (0.39, 0.50)	<0.001

Data source: National Center for Health Statistics, National Health Interview Survey, July 2020–December 2021

In the full model (model 3), among the predisposing factors, the negative association of Asian race on the odds of having a telemedicine visit remained (aOR: 0.75, *p*<0.001). However, Hispanic ethnicity was no longer a significant association. In relation to age, only being in the oldest group (50–64 years), compared with being 18–29 years, significantly lowered the odds, while being female increased the odds. All three need factors were associated with increased odds of having a telehealth visit: being in fair/poor health, having a disability, and having ≥1 chronic condition(s). Among the enabling factors, both education and family income followed a gradient; lower education levels and income were associated with lower odds of a telemedicine visit. Compared to being privately insured, public or other insurance increased the odds, while being uninsured lowered the odds. Not having a usual place of care and living in nonmetropolitan areas were associated with lower odds of a telemedicine visit.

### Factors Associated with ED and Hospital Visits

We tested whether English proficiency and telemedicine were associated with having any ED and hospital visits. We found that limited English proficiency was not significantly associated with either type of visit. Having any telemedicine visit did not modify the effect (Wald’s *χ*^2^ test of joint significance, both *p*>0.05). However, having a telemedicine visit was independently associated with significantly greater odds of having an ED (aOR: 1.80, *p*<0.001) and a hospital visit (aOR: 2.03, *p*<0.001) (Table 3). Full models are available in Appendix B.

**Table 3:** Multivariable Associations with Healthcare Utilization Among Nonelderly US Adults, National Health Interview Survey, July 2020–December 2021

	Any emergency department visits		Any hospital visits	
	aOR (95% CI)	*p*-value	aOR (95% CI)	*p*-value
English proficiency				
English-proficient	Ref.		Ref.	
Limited English-proficient	0.86 (0.71, 1.05)	0.15	0.85 (0.63, 1.15)	0.29
Telemedicine visit				
No	Ref.		Ref.	
Yes	1.80 (1.64, 1.97)	<0.001	2.03 (1.79, 2.31)	<0.001

Data source: National Center for Health Statistics, National Health Interview Survey, July 2020–December 2021

Models are adjusted for age, sex, race/ethnicity, health status, has a disability, has ≥1 chronic condition, education, family income, insurance, location, and has a usual place of care

### Sensitivity Analyses

We tested the interaction between English proficiency and race and ethnicity in the ED and hospital visit models. The interactions were not statistically significant (Wald’s *χ*^2^ test of joint significance, both *p*>0.05), and we concluded the effect of English proficiency on healthcare utilization was not modified by race and ethnicity.

## DISCUSSION

During the COVID-19 pandemic, a large portion of US adults used telemedicine to access healthcare. Using a nationally representative sample of nonelderly adults, we found that between July 2020 and December 2021, 22% of adults with LEP had a telemedicine visit compared to 35% of adults with EP. While these rates of telemedicine use are markedly higher than pre-pandemic rates in both groups (e.g., 5% of LEP and 12% of EP in California used telemedicine between 2015 and 2018),^19^ as hypothesized, this study found adults with LEP to be 20% less likely than adults with EP to have a telemedicine visit, even after accounting for predisposing, enabling, and need factors.

Prior studies examining telemedicine use in California, Oregon, Pennsylvania, and New Jersey have reported similar or larger disparities in telemedicine use between LEP and EP patients, both before and after the start of the pandemic.^17–19^ Notably, in our national study, we observed that between July 2020 and December 2021, telemedicine visit rates among adults with LEP remained virtually unchanged while telemedicine visit rates increased among adults with EP. If this pattern persists, the disparity in telemedicine use between LEP and EP adults will worsen if nothing is done to mitigate it.

The disparity in telemedicine use among adults with LEP likely reflects a confluence of structural barriers to accessing telemedicine.^15,32^ In addition to being impacted by the digital divide, LEP adults also encounter LEP-specific barriers, including the need for medical interpreters and English-only virtual platforms and technologies.^15,21,32^ To improve access to telemedicine, healthcare systems need to be intentional in designing and implementing services to be accessible to all patients. Successful strategies to address disparities among LEP patients include interventions to address healthcare system barriers, such as custom building patient portals in the most frequent patient languages, using virtual platforms that do not require application download or patient portal signup, ensuring easy inclusion of interpreters in telemedicine visits, and partnering with local organizations to identify and address language and culture-specific needs.^15,32^ Patient-centered strategies may include outreach and education in multiple languages to help patients signup for patient portals and how to set up and conduct telemedicine visits on multiple device types.^15,32^

Consistent with findings from previous studies,^17–19^ we also found Asian race to be associated with a lower likelihood of telemedicine use, after controlling for English proficiency. Despite higher rates of internet use and technology adoption reported among English-speaking Asian Americans,^33^ our study findings concerning telemedicine use mirror early findings of lower patient portal adoption rates among Asian Americans.^34^ Less use of telemedicine may reflect lower overall healthcare use,^35–37^ negative experiences with the healthcare system,^38^ and even differences in where they seek care.^39,40^ Whether the difference in telemedicine use among Asian Americans can be explained by differences in preferences, experiences, or other factors is unclear and requires further investigation.

We found that adults with LEP and EP have a similar likelihood of using the ED and hospital, after adjusting for covariates, despite a larger percentage of adults with LEP having any ED visits. Also, contrary to our hypothesis, we did not find that the interaction between English proficiency and telemedicine use moderated the relationship. Previous works examining differences in ED and hospital use among adults with LEP have been mixed.^8,41,42^ One MEPS-based study found Hispanic LEP adults to be associated with lower rates of ED and hospital visits than both Hispanic and non-Hispanic EP adults,^8^ while one health system–based study found LEP adults to have higher rates of ED and hospital visits than EP adults.^41,42^ From our findings, we conclude that English proficiency was not associated with an excess of ED visits, though we cannot determine whether the amount of healthcare used was clinically appropriate. Coupling our findings with those of a previous study showing that a greater percentage of adults with LEP delayed or forwent healthcare during the pandemic^10^ cautions that the lack of difference in ED visits between adults with LEP and EP may be temporary. Continued vigilance in monitoring the level and types of healthcare utilization among adults with LEP is needed to ensure that telemedicine does not result in disparities in access and use.

While telemedicine did not moderate the relationship between English proficiency and ED and hospital visits, we found telemedicine use to be positively associated with any ED and hospital visits. The relationship between telemedicine use and other healthcare utilization has been found to vary by reason for the visit, telemedicine mode, and care setting.^23–26,43^ While the NHIS data does not allow us to differentiate between telemedicine modes (i.e., video, telephone), care settings, sequence, or reasons for telemedicine visits, our findings are similar to another cross-sectional study that found telemedicine use to be positively associated with ED visits.^19^ Given the cross-sectional nature of the data, the extent to which telemedicine use led to increases in ED and hospital visits or the reverse is unclear. On the one hand, telemedicine visits may have caused more ED visits, perhaps due to a greater prevalence of telephone visits or telemedicine visits for acute conditions during the pandemic.^24,43^ One claims-based study found that, compared to in-person encounters, telemedicine encounters for acute conditions were more likely to lead to follow-up ED visits while follow-up visits were similar for telemedicine encounters for chronic conditions.^24^ Another study of primary care visits in Northern California found ED visits to be higher after primary care telephone visits (but not video visits).^43^ Conversely, telemedicine visits may have facilitated healthcare access by allowing more post-discharge follow-up visits to be conducted virtually, reducing traditional barriers such as transportation issues and COVID-19-driven barriers like fears of infection.^20,25,26^ A study of hospitals in Pennsylvania and a study of primary care clinics in New York City both found telemedicine visits to increase follow-up primary care visits.^25,26^ Further research is needed to elucidate the relationship between telemedicine and downstream healthcare utilization.

The telemedicine policy landscape continues to evolve. Many of the flexibilities supporting telemedicine coverage and payment, enacted by the federal government to address the COVID public health emergency, expire at the end of 2024.^44^ Private payers and many states are expected to follow suit, resulting in a more limited scope and reach of telemedicine services.

On the other hand, other changes made during the pandemic suggest that telemedicine will persist as a form of healthcare delivery. Many healthcare systems made substantial investments in telehealth, including infrastructure and staff training. Additionally, many patients prefer telemedicine, particularly for routine medical care and ongoing mental health services.^45^ Also, while many of the federal government’s healthcare-specific investments may be ending, its continuing support for infrastructure improvements to bring high-speed internet access to both urban and rural hard-to-reach areas will expand the potential telemedicine patient population.^46^ Coupled with provider shortages, telemedicine may stand out as a financially advantageous avenue for organizing care delivery, namely care from providers who are fluent in languages other than English.

Our study has several limitations. First, the 2020 and 2021 NHIS did not ask respondents how well they spoke English, so we based our definition of LEP on the language in which the survey was administered. While this approach may not have accurately captured respondents’ ability to speak and understand English, interview language is a common proxy for English proficiency in the health services literature because respondents interviewing in another language have been found to commonly need healthcare language services.^7–9,47^ Second, data were obtained from patient self-report and not confirmed through medical records; some variables may be subject to recall bias.^48^ Finally, the recall period for utilization measures was the previous 12 months so the earlier cohorts included a period both before the February 2020 declaration of a public health emergency and during the pandemic. Despite limitations, our study confirms at a national scale, the earlier and more geographically limited findings of key disparities in telemedicine use among LEP adults. These findings support pursuing further investigation to better understand the interplay of reasons for disparities in telemedicine use.

## CONCLUSIONS

Nationally, adults with LEP reported being less likely to use telemedicine than other adults during the first 1.5 years of the COVID-19 pandemic. This disparity persisted after controlling for predisposing, need, and enabling factors that can affect access. Use of telemedicine was associated with having ED and hospital visits. These findings highlight that if policymakers support the uptake of telemedicine as a viable and important avenue to care, attention needs to focus on interventions addressing access barriers based on language to ensure adults with LEP are not left behind in the digital divide.

### Supplementary Information﻿

Below is the link to the electronic supplementary material.

Supplementary file1 (DOCX 31.3 KB)

## Data Availability

Data from 2020 and 2021 were downloaded from the CDC NHIS website (https://www.cdc.gov/nchs/nhis/index.htm).
