# Supplementary material for: Differences in Telemedicine, Emergency Department, and Hospital Utilization Among Nonelderly Adults with Limited English Proficiency Post-COVID-19 Pandemic: a Cross-Sectional Analysis
Source: J Gen Intern Med. 2023 Aug 17;38(16):3490–8. doi: 10.1007/s11606-023-08353-7 (PMC10713935; doi:10.1007/s11606-023-08353-7)
Supplement: Supplementary file 1 — Supplementary file1 (DOCX 31.3 KB) [file 11606_2023_8353_MOESM1_ESM.docx]

**Supplementary Information**

Appendix A. National Health Interview Survey Questions, 2020 and 2021

| Outcome | Description | Question text | Population |
| --- | --- | --- | --- |
|  |  |  |  |
| Any telemedicine visits | Virtual medical appointment, past 12m | In the past 12 months, have you had an appointment with a doctor, nurse, or other health professional by video or by phone? | Sample adults 18+ |
| Any emergency department visits | Number of times visited hospital emergency room, past 12m, top-coded | During the past 12 months, how many times have you gone to a hospital emergency room about your health? | Sample adults 18+ |
| Any hospital visits | Hospitalized overnight, past 12m | During the past 12 months, have you been hospitalized overnight? | Sample adults 18+ |

Appendix B.1. Multivariable models for any emergency department visits among nonelderly US adults, National Health Interview Survey, July 2020-December 2021*

|  | **Any emergency department visits** | | | | | | | |
| --- | --- | --- | --- | --- | --- | --- | --- | --- |
|  | Model 1 | |  | Model 2 | |  | Model 3 | |
|  | aOR (95% CI) | p-value |  | aOR (95% CI) | p-value |  | aOR (95% CI) | p-value |
| English proficiency |  |  |  |  |  |  |  |  |
| English proficient | ref. |  |  | ref. |  |  | ref. |  |
| Limited English proficient | 0.84 (0.69, 1.03) | 0.10 |  | 0.86 (0.71, 1.05) | 0.15 |  | 0.87 (0.69, 1.10) | 0.25 |
| Telemedicine visit |  |  |  |  |  |  |  |  |
| No | -- | -- |  | ref. |  |  | ref. | ref. |
| Yes | -- | -- |  | 1.80 (1.64, 1.97) | <0.001 |  | 1.80 (1.64, 1.97) | <0.001 |
| English proficiency*telemedicine visit | -- | -- |  | -- | -- |  | 0.98 (0.67, 1.42) | 0.91 |
| **Predisposing Factors** |  |  |  |  |  |  |  |  |
| Age |  |  |  |  |  |  |  |  |
| 18-29 | ref. |  |  | ref. |  |  | ref. | ref. |
| 30-39 | 0.87 (0.77, 0.99) | 0.04 |  | 0.87 (0.77, 0.98) | 0.02 |  | 0.87 (0.77, 0.98) | 0.02 |
| 40-49 | 0.68 (0.60, 0.78) | <0.001 |  | 0.68 (0.59, 0.78) | <0.001 |  | 0.68 (0.59, 0.78) | <0.001 |
| 50-64 | 0.66 (0.58, 0.75) | <0.001 |  | 0.67 (0.59, 0.77) | <0.001 |  | 0.67 (0.59, 0.77) | <0.001 |
| Sex |  |  |  |  |  |  |  |  |
| Male | ref. |  |  | ref. |  |  | ref. | ref. |
| Female | 1.32 (1.21, 1.43) | <0.001 |  | 1.23 (1.13, 1.34) | <0.001 |  | 1.23 (1.13, 1.34) | <0.001 |
| Race/ethnicity |  |  |  |  |  |  |  |  |
| Non-Hispanic White | ref. |  |  | ref. |  |  | ref. | ref. |
| Hispanic | 1.13 (0.99, 1.28) | 0.07 |  | 1.13 (1.00, 1.29) | 0.05 |  | 1.13 (1.00, 1.29) | 0.05 |
| Non-Hispanic Asian | 0.61 (0.51, 0.74) | <0.001 |  | 0.63 (0.52, 0.77) | <0.001 |  | 0.63 (0.52, 0.77) | <0.001 |
| **Need Factors** |  |  |  |  |  |  |  |  |
| Health status |  |  |  |  |  |  |  |  |
| Excellent/very good/good | ref. |  |  | ref. |  |  | ref. | ref. |
| Poor/fair | 2.22 (1.96, 2.52) | <0.001 |  | 2.06 (1.82, 2.34) | <0.001 |  | 2.06 (1.82, 2.34) | <0.001 |
| Has a disability |  |  |  |  |  |  |  |  |
| No | ref. |  |  | ref. |  |  | ref. | ref. |
| Yes | 1.35 (1.15, 1.58) | <0.001 |  | 1.26 (1.08, 1.48) | 0.004 |  | 1.26 (1.08, 1.48) | 0.004 |
| Has ≥1 chronic condition |  |  |  |  |  |  |  |  |
| No | ref. |  |  | ref. |  |  | ref. | ref. |
| Yes | 1.47 (1.33, 1.63) | <0.001 |  | 1.36 (1.23, 1.51) | <0.001 |  | 1.36 (1.23, 1.51) | <0.001 |
| **Enabling Factors** |  |  |  |  |  |  |  |  |
| Education |  |  |  |  |  |  |  |  |
| Some college or more | ref. |  |  | ref. |  |  | ref. | ref. |
| High school diploma | 1.21 (1.09, 1.34) | <0.001 |  | 1.27 (1.15, 1.41) | <0.001 |  | 1.27 (1.15, 1.41) | <0.001 |
| Less than high school diploma | 1.22 (1.01, 1.47) | 0.04 |  | 1.31 (1.09, 1.58) | 0.005 |  | 1.31 (1.09, 1.58) | 0.005 |
| Family income (% FPL) |  |  |  |  |  |  |  |  |
| ≥200% | ref. |  |  | ref. |  |  | ref. | ref. |
| 100-199% | 1.24 (1.09, 1.40) | <0.001 |  | 1.26 (1.12, 1.43) | <0.001 |  | 1.26 (1.12, 1.43) | <0.001 |
| <100% | 1.25 (1.09, 1.45) | 0.002 |  | 1.29 (1.12, 1.49) | <0.001 |  | 1.29 (1.12, 1.49) | <0.001 |
| Insurance |  |  |  |  |  |  |  |  |
| Private | ref. |  |  | ref. |  |  | ref. | ref. |
| Public/Other | 1.60 (1.42, 1.81) | <0.001 |  | 1.56 (1.39, 1.76) | <0.001 |  | 1.56 (1.39, 1.76) | <0.001 |
| Uninsured | 1.03 (0.88, 1.20) | 0.71 |  | 1.11 (0.95, 1.30) | 0.18 |  | 1.11 (0.95, 1.30) | 0.18 |
| Location |  |  |  |  |  |  |  |  |
| Metropolitan | ref. |  |  | ref. |  |  | ref. | ref. |
| Nonmetropolitan | 1.16 (1.04, 1.30) | 0.008 |  | 1.24 (1.11, 1.39) | <0.001 |  | 1.24 (1.11, 1.39) | <0.001 |
| Usual place of care |  |  |  |  |  |  |  |  |
| Yes | ref. |  |  | ref. |  |  | ref. | ref. |
| No | 1.09 (0.95, 1.25) | 0.22 |  | 1.18 (1.03, 1.36) | 0.02 |  | 1.18 (1.03, 1.36) | 0.02 |

Data Source: National Center for Health Statistics, National Health Interview Survey, July 2020-December 2021

* Model 1 is the fully adjusted model without having a telemedicine visit. Model 2 is the fully adjusted model with having a telemedicine visit. Model 3 is the fully adjusted model with an interaction term between English proficiency and having a telemedicine visit.

Appendix B.2. Multivariable models for any hospital visits among nonelderly US adults, National Health Interview Survey, July 2020-December 2021*

|  | **Any hospital visits** | | | | | | | |
| --- | --- | --- | --- | --- | --- | --- | --- | --- |
|  | Model 1 | |  | Model 2 | |  | Model 3 | |
|  | aOR (95% CI) | p-value |  | aOR (95% CI) | p-value |  | aOR (95% CI) | p-value |
| English proficiency |  |  |  |  |  |  |  |  |
| English proficient | ref. |  |  | ref. |  |  | ref. |  |
| Limited English proficient | 0.82 (0.61, 1.11) | 0.20 |  | 0.85 (0.63, 1.15) | 0.29 |  | 0.85 (0.59, 1.22) | 0.37 |
| Telemedicine visit |  |  |  |  |  |  |  |  |
| No | -- | -- |  | ref. |  |  | ref. |  |
| Yes | -- | -- |  | 2.04 (1.79, 2.32) | <0.001 |  | 2.03 (1.78, 2.32) | <0.001 |
| English proficiency*telemedicine visit | -- | -- |  | -- | -- |  | 1.01 (0.60, 1.71) | 0.96 |
| **Predisposing Factors** |  |  |  |  |  |  |  |  |
| Age |  |  |  |  |  |  |  |  |
| 18-29 | ref. |  |  | ref. |  |  | ref. |  |
| 30-39 | 1.13 (0.93, 1.38) | 0.21 |  | 1.13 (0.93, 1.37) | 0.23 |  | 1.13 (0.93, 1.38) | 0.23 |
| 40-49 | 0.73 (0.59, 0.89) | 0.003 |  | 0.73 (0.59, 0.89) | 0.003 |  | 0.73 (0.59, 0.89) | 0.003 |
| 50-64 | 0.89 (0.73, 1.08) | 0.24 |  | 0.92 (0.75, 1.11) | 0.38 |  | 0.92 (0.75, 1.11) | 0.38 |
| Sex |  |  |  |  |  |  |  |  |
| Male | ref. |  |  | ref. |  |  | ref. |  |
| Female | 1.79 (1.59, 2.02) | <0.001 |  | 1.66 (1.46, 1.88) | <0.001 |  | 1.66 (1.46, 1.88) | <0.001 |
| Race/ethnicity |  |  |  |  |  |  |  |  |
| Non-Hispanic White | ref. |  |  | ref. |  |  | ref. |  |
| Hispanic | 1.06 (0.89, 1.25) | 0.54 |  | 1.06 (0.89, 1.26) | 0.51 |  | 1.06 (0.89, 1.26) | 0.51 |
| Non-Hispanic Asian | 0.68 (0.51, 0.90) | 0.008 |  | 0.70 (0.53, 0.94) | 0.02 |  | 0.70 (0.53, 0.94) | 0.02 |
| **Need Factors** |  |  |  |  |  |  |  |  |
| Health status |  |  |  |  |  |  |  |  |
| Excellent/very good/good | ref. |  |  | ref. |  |  | ref. |  |
| Poor/fair | 2.40 (2.00, 2.88) | <0.001 |  | 2.17 (1.81, 2.61) | <0.001 |  | 2.17 (1.81, 2.61) | <0.001 |
| Has a disability |  |  |  |  |  |  |  |  |
| No | ref. |  |  | ref. |  |  | ref. |  |
| Yes | 1.57 (1.27, 1.94) | <0.001 |  | 1.46 (1.19, 1.80) | <0.001 |  | 1.46 (1.19, 1.80) | <0.001 |
| Has ≥1 chronic condition |  |  |  |  |  |  |  |  |
| No | ref. |  |  | ref. |  |  | ref. |  |
| Yes | 1.85 (1.60, 2.14) | <0.001 |  | 1.69 (1.46, 1.95) | <0.001 |  | 1.69 (1.46, 1.95) | <0.001 |
| **Enabling Factors** |  |  |  |  |  |  |  |  |
| Education |  |  |  |  |  |  |  |  |
| Some college or more | ref. |  |  | ref. |  |  | ref. |  |
| High school diploma | 1.13 (0.99, 1.29) | 0.08 |  | 1.20 (1.04, 1.38) | 0.01 |  | 1.20 (1.04, 1.38) | 0.01 |
| Less than high school diploma | 1.08 (0.84, 1.39) | 0.54 |  | 1.18 (0.92, 1.52) | 0.20 |  | 1.18 (0.92, 1.52) | 0.20 |
| Family income (% FPL) |  |  |  |  |  |  |  |  |
| ≥200% | ref. |  |  | ref. |  |  | ref. |  |
| 100-199% | 1.24 (1.03, 1.49) | 0.02 |  | 1.27 (1.06, 1.53) | 0.01 |  | 1.27 (1.06, 1.53) | 0.01 |
| <100% | 1.17 (0.94, 1.46) | 0.16 |  | 1.21 (0.97, 1.51) | 0.09 |  | 1.21 (0.97, 1.51) | 0.09 |
| Insurance |  |  |  |  |  |  |  |  |
| Private | ref. |  |  | ref. |  |  | ref. |  |
| Public/Other | 1.37 (1.15, 1.63) | <0.001 |  | 1.32 (1.11, 1.58) | 0.002 |  | 1.32 (1.11, 1.58) | 0.002 |
| Uninsured | 0.85 (0.65, 1.12) | 0.25 |  | 0.95 (0.73, 1.25) | 0.72 |  | 0.95 (0.73, 1.25) | 0.73 |
| Location |  |  |  |  |  |  |  |  |
| Metropolitan | ref. |  |  | ref. |  |  | ref. |  |
| Nonmetropolitan | 1.16 (0.99, 1.37) | 0.06 |  | 1.27 (1.08, 1.48) | 0.004 |  | 1.27 (1.08, 1.48) | 0.004 |
| Usual place of care |  |  |  |  |  |  |  |  |
| Yes | ref. |  |  | ref. |  |  | ref. |  |
| No | 0.72 (0.57, 0.91) | 0.007 |  | 0.81 (0.63, 1.02) | 0.08 |  | 0.81 (0.63, 1.02) | 0.08 |

Data Source: National Center for Health Statistics, National Health Interview Survey, July 2020-December 2021

* Model 1 is the fully adjusted model without having a telemedicine visit. Model 2 is the fully adjusted model with having a telemedicine visit. Model 3 is the fully adjusted model with an interaction term between English proficiency and having a telemedicine visit.
